# It's not just the mitral valve - abnormal motion of the whole aorto-mitral apparatus occurs in both overt and subclinical hypertrophic cardiomyopathy

**DOI:** 10.1186/1532-429X-18-S1-Q37

**Published:** 2016-01-27

**Authors:** Chinwe Obianyo, Gaby Captur, Petros Syrris, Luis Lopes, Vimal Patel, Patricia Reant, Mariana Mirabel, Charlotte Manisty, Paul Bassett, Daniel Sado, Phillippa Talmud, Perry M Elliott, James C Moon

**Affiliations:** 1 grid.83440.3b0000000121901201Institute of Cardiovascular Science, University College London, London, United Kingdom; 2 Bart's Heart Centre and Centre for Biostatistics and Epidemiology, London, United Kingdom; 3 grid.9983.b0000000121814263Cardiovascular centre, University of Lisbon, Lisbon, Portugal; 4 grid.42399.350000000405937118University of Bordeaux, CHU de Bordeaux, Bordeaux, France; 5 grid.414093.bAssistance Publique-Hôpitaux de Paris, Hôpital Européen Georges Pompidou, Paris, France; 6 Cardiology Department Kings College, London, United Kingdom

## Background

Mitral valve abnormalities are an important cause of patient morbidity in hypertrophic cardiomyopathy (HCM) contributing to left ventricular outflow tract obstruction (LVOTO). Anterior mitral valve leaflet (AMVL) elongation predisposes to LVOTO. CMR detects this in overt HCM and in sarcomere gene mutation carriers without left ventricular hypertrophy (G+LVH-). However, the geometry of the mitral valve, the subvalvular apparatus and the LVOT is more complex. The purpose of this study was analyse the whole aorto-mitral area (the aorto-mitral apparatus) and track its dynamic motion to explore mechanisms involved in LVOTO-predisposition in subclinical HCM.

## Methods

**Figure 1:**
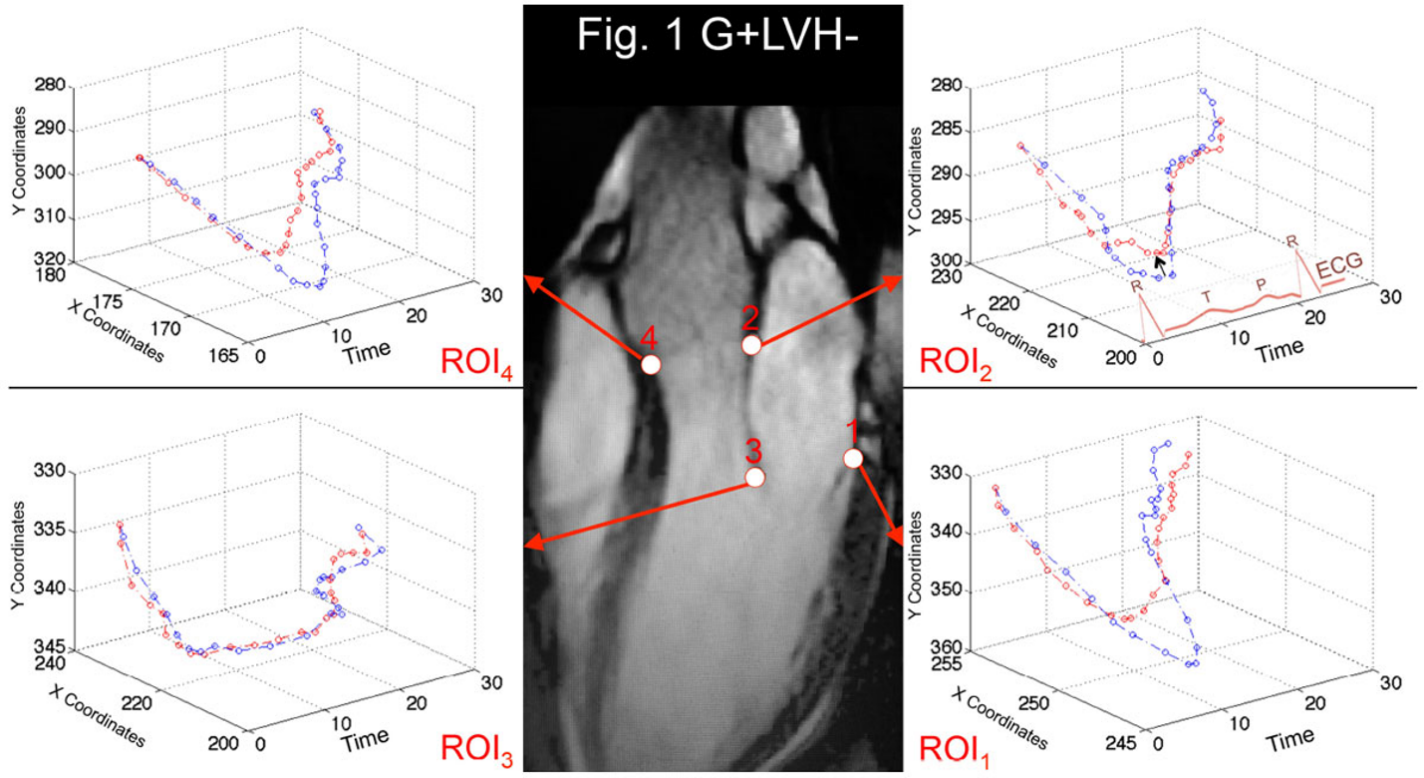
**2D displacement-versus-time plots in G+LVH- (red) versus matched healthy-volunteers (blue) normalised for RR intervals and baseline positions**. Lines represent population means. Motion tracking software (author, GC) interactively measured the x/y coordinates in 2D space and at multiple time points gated to the ECG R-R interval for each of the 4 ROIs during the cardiac cycle. On the ‘Time' axis, the first ECG R wave represents start time t = 0. Black arrow points to the presence of subtle but statistically significant SAM of the intertrigonal mitral annulus in subclinical HCM.

CMR using a 1.5-tesla scanner (Avanto, Siemens) was performed on 35 G+LVH- patients without left atrial (LA) enlargement (31 ± 14 years, 34% male), 31 patients with a clinical diagnosis of HCM with preserved ejection fraction (47 ± 12 years, 61% male, all with pathogenic/likely pathogenic sarcomere mutations [G+LVH+]), and 53 matched healthy volunteers (45% male, 42 ± 14). Direct assessment of the aortomitral apparatus was performed on the 3-chamber view acquired using a breath-held steady-state free precession sequence. Cines were interrogated frame-by-frame, semi-automatically using an in-house script developed for MATLAB®. The motion tracking software interactively assigned, tracked and graphed, the dynamic excursion of 4 pre-defined aortomitral regions of interest (ROI) throughout one cardiac cycle: ROI_1_ - hinge point of the posterior MVL; ROI_2_ - intertrigonal mitral annulus; ROI_3_ - tip of the AMVL; ROI_4_ - anterior aortic annulus (Fig. 1).

## Results

**Figure 2:**
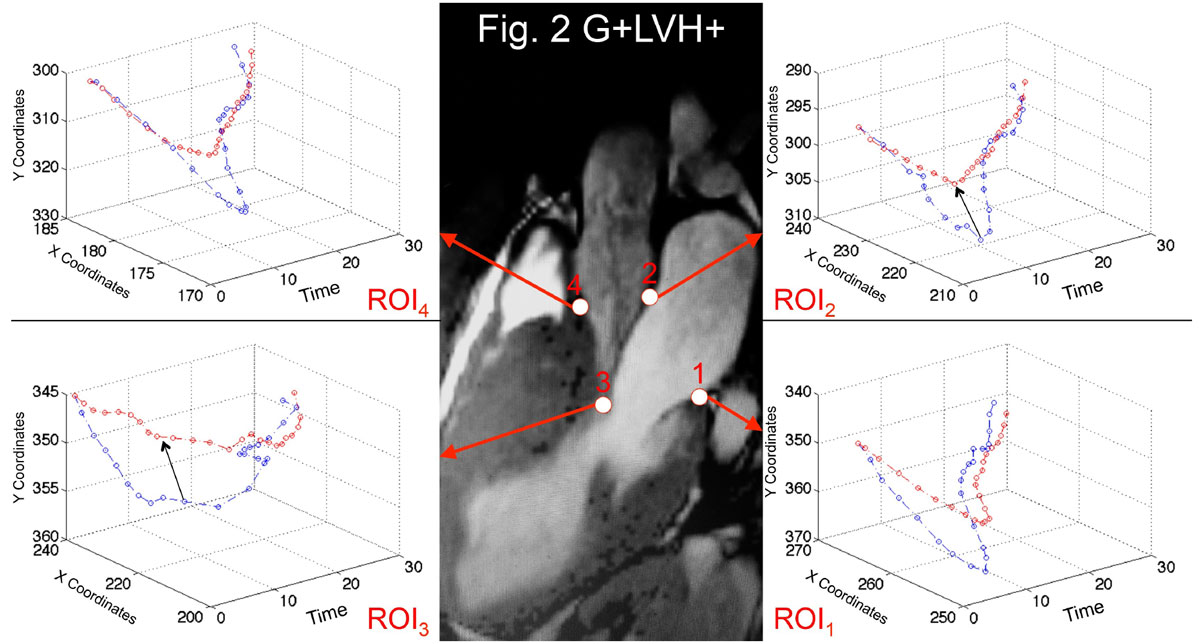
**Normalised 2D displacement-versus-time plots in G+LVH+ (red) versus matched healthy volunteers (blue)**. Lines represent population means. Black arrows point to the presence of SAM in overt HCM.

Qualitative examination of normalized two-dimensional displacement-versus-time plots in G+LVH- patients revealed subtle systolic anterior motion (SAM) of the intertrigonal mitral annulus and reduced longitudinal excursion compared to controls. Statistically significant differences were present for ROI_1_ (*P* = 0.005), ROI_2_ (*P* = 0.01) and ROI_4_ (*P* < 0.0001, Fig. 1). In G+LVH+ patients, displacement-versus-time plots for all 4 ROIs differed significantly from those of controls resulting from a combination of SAM, diminished longitudinal excursion and blunted end-diastolic motion (Fig. 2): ROI_1_ (*P* = 0.02), ROI_2_ (*P* = 0.007), ROI_3_ (*P* < 0.0001) and ROI_4_ (*P* < 0.0001).

## Conclusions

The AMVL is longer in preclinical HCM, but in addition, there are more widespread abnormalities of the aorto-mitral apparatus motion, including SAM of the mitral annulus before the development of LVH or LA enlargement. These data have the potential to improve our understanding of early phenotype development and LVOTO-predisposition in HCM.

